# Prognostic value of neutrophil-to-lymphocyte ratio in COVID-19 compared with Influenza and respiratory syncytial virus infection

**DOI:** 10.1038/s41598-021-00927-x

**Published:** 2021-11-02

**Authors:** Lior Prozan, Eden Shusterman, Jacob Ablin, Alexis Mitelpunkt, Ahuva Weiss-Meilik, Amos Adler, Guy Choshen, Orli Kehat

**Affiliations:** 1 Department of Internal Medicine H, Tel Aviv Medical Center, 6 Weizmann St., 64239 Tel Aviv, Israel; 2 I-Medata AI Center, Tel Aviv Medical Center, 6 Weizmann St., 64239 Tel Aviv, Israel; 3 Sackler Faculty of Medicine, Tel Aviv University, Ramat Aviv, Tel Aviv, Israel; 4 Infectious Diseases Unit, Tel Aviv Medical Center, 6 Weizmann St., 64239 Tel Aviv, Israel; 5 Pediatric Rehabilitation Service, “Dana-Dwek” Children’s Hospital, Tel Aviv Medical Center, 6 Weizmann St., 64239 Tel Aviv, Israel; 6 Microbiology Laboratory, Tel Aviv Medical Center, 6 Weizmann St., 64239 Tel Aviv, Israel

**Keywords:** Infectious diseases, Prognostic markers

## Abstract

A high neutrophil to lymphocyte ratio (NLR) is considered an unfavorable prognostic factor in various diseases, including COVID-19. The prognostic value of NLR in other respiratory viral infections, such as Influenza, has not hitherto been extensively studied. We aimed to compare the prognostic value of NLR in COVID-19, Influenza and Respiratory Syncytial Virus infection (RSV). A retrospective cohort of COVID-19, Influenza and RSV patients admitted to the Tel Aviv Medical Center from January 2010 to October 2020 was analyzed. Laboratory, demographic, and clinical parameters were collected. Two way analyses of variance (ANOVA) was used to compare the association between NLR values and poor outcomes among the three groups. ROC curve analyses for each virus was applied to test the discrimination ability of NLR. 722 COVID-19, 2213 influenza and 482 RSV patients were included. Above the age of 50, NLR at admission was significantly lower among COVID-19 patients (P < 0.001). NLR was associated with poor clinical outcome only in the COVID-19 group. ROC curve analysis was performed; the area under curve of poor outcomes for COVID-19 was 0.68, compared with 0.57 and 0.58 for Influenza and RSV respectively. In the COVID-19 group, multivariate logistic regression identified a high NLR (defined as a value above 6.82) to be a prognostic factor for poor clinical outcome, after adjusting for age, sex and Charlson comorbidity score (odds ratio of 2.9, P < 0.001). NLR at admission is lower and has more prognostic value in COVID-19 patients, when compared to Influenza and RSV.

## Introduction

Although most patients infected with severe acute respiratory syndrome coronavirus 2 (SARS-CoV-2) are either asymptomatic or else undergo no more than a mild febrile course, a distinct minority will suffer a severe systemic course, the hallmark of which is the development of respiratory failure.

Immune dysregulation and damage to lymphocytes, especially T-lymphocytes, have been observed among Coronavirus disease 2019 (COVID-19) patients^1^. Increased serum levels of proinflammatory cytokines (TNF-α, IL-1, and IL-6) and chemokines (IL-8) have been found in patients with severe COVID-19, compared with individuals with mild disease, suggesting a possible role for a hyperinflammatory response in the pathogenesis of COVID-19^1,2^.

Neutrophil-to-lymphocyte ratio (NLR) is a parameter used to assess the inflammatory status of a subject. It can easily be calculated from a complete blood count, a commonly used blood test^3^. NLR has previously been shown to predict outcomes in a variety of conditions including different types of malignancies, as well as in cardiovascular and rheumatic diseases^4–9^. Studies have also shown NLR to be useful in predicting outcomes of different types of infections, including community acquired pneumonia, bacteremia and endocarditis^10–12^. NLR has been studied and proved to be useful in differentiating between patients hospitalized with fever due to infection and those with fever due to non-infectious causes^13,14^.

Focusing on viral infections (non COVID-19), the application of NLR has rarely been reported. One study showed the prognostic value of NLR in patients infected with avian influenza^15^. A number of recently published studies have found NLR to have a sensitivity of 70–86% in detecting influenza virus (A and B) infection in both elderly and pediatric populations^16–18^. However, these studies were small scale, including up to 100 patients in the case group, and up to 225 patients in the control group.

Many studies have recognized increased NLR as an independent prognostic factor for COVID-19 patients, especially in those older than 50. Four meta-analyses have demonstrated that an increased NLR can serve as an early warning signal of severe COVID-19^1,19–31^. The criteria for severe disease varied between each study, but most studies considered the illness to be severe if a patient presented with one of the following: Respiratory distress, with a respiratory rate (RR) ≥ 30 breaths/min; Oxygen saturation (SpO2) ≤ 93% in the resting state; Arterial blood oxygen partial pressure (PaO_2_)/oxygen concentration (FiO_2_) (PF ratio) ≤ 300 mmHg^22^. Furthermore, NLR was found to be a prognostic factor for endotracheal intubation upon hospital admission and an independent predictor for risk of all-cause mortality during hospitalization^20,29^. The proposed optimum cut-off values to distinguish high NLR from low NLR ranged from 3 to 6^25^.

One recently published very small scale study from Taiwan investigated NLR as a marker for likelihood of COVID-19, in order to distinguish the disease from other upper respiratory infections (URI) in the early phase^32^. However, in their analysis, NLR elevation showed no difference between COVID-19 and URI.

Influenza virus and respiratory syncytial virus are the two major viral pathogens associated with acute lower respiratory infection^33^. Several reports have described the global seasonality of these viruses. In these studies, both influenza and respiratory syncytial virus circulation peaks were well aligned with winter months in temperate regions^34–37^. Therefore, during the upcoming winter, clinicians worldwide will need to make fast and evidence-based decisions regarding further evaluation and treatment of patients presenting with flu-like symptoms.

Thus, in the current study, we aim to compare the levels and prognostic value of NLR in COVID-19 with two other common respiratory viruses: Influenza virus (A and B) and respiratory syncytial virus (RSV).

## Methods

### Study design

We conducted a retrospective observational study at the Tel Aviv Sourasky Medical Center, a tertiary-level 1200-bed academic hospital. For data collection purposes, we searched the microbiology laboratory database for positive reverse transcription polymerase chain reaction (RT-PCR) nasal swabs for Influenza (A and B), RSV and SARS-CoV-2 from 2010 through 2020. Patient’s data were extracted from electronic medical charts. The STROBE checklist was followed for all study procedures^38^. The study was reviewed and approved by the Tel Aviv Sourasky Medical Center institutional review board. All methods were performed in accordance with the relevant guidelines and regulations. The institutional review board that approved the study also waived the need for informed consent due to the retrospective observational nature of the study.

### Patients

Patients were selected according to the following criteria: 1. Positive viral PCR for SARS-CoV-2, influenza (A or B) or RSV; 2. Hospital admission from 1.1.2010 to 7.10.2020; 3. Documented complete blood count (CBC). We did not include patients with co-infection of SARS-CoV-2 or influenza (A or B) or RSV. Only patients over the age of 18 were included.

### Data collection

Data were extracted from patient’s electronic health records. Baseline patient information included age, sex, and comorbidities, summarized using the Charlson comorbidity index^39^. For each patient, vital signs at admission (body temperature, heart rate, blood pressure, oxygen saturation), laboratory data (white blood cell and differential blood counts, C-reactive protein, ferritin, triglycerides) were retrieved. NLR values were calculated by dividing neutrophil count by lymphocyte count at admission. The primary outcome measure was a composite score of poor outcomes consisting of mortality, defined as death at 30 days and need for mechanical ventilation.

### Microbiology

The diagnosis of viral respiratory infection was done by PCR. Samples were collected by combined pharyngeal and nasopharyngeal swabs that were inoculated into UTM tubes and immediately transported to the laboratory. RNA extraction was done using the easyMAG® system (BioMérieux, Marcy-l'Étoile, France). The diagnosis of SARS-CoV-2 was done by RT-PCR using mostly the Allplex™ 2019-nCoV Assay (Seegene). The diagnosis of Influenza A&B and RSV was done using the Simplexa™ Flu A/B & RSV kit (DiaSorin) or the Seeplex® RV7 kit (Seegene).

### Statistical analyses

The characteristics of patients with influenza, RSV or COVID-19 were summarized as medians and interquartile ranges for continues variables and counts and percentages for categorical variables. Kruskal–Wallis and Pearson’s chi-square tests were used to compare between the three respiratory infections. For comparison between NLR values, adverse outcomes and their interaction among the three infection groups, two-way analysis of variance (ANOVA) was used. Since the distribution of NLR was highly skewed, log transformation was applied to create normally distributed data. The predictive values of NLR for poor clinical outcome were assessed using the receiver operating characteristic (ROC) curve. For COVID-19, the optimal cutoff value of NLR was determines using Youden’s Index. Multivariate logistic regression was further performed to obtain the odds ratio (OR) of NLR and additional factors. Statistical calculations were performed using the SPSS 25.0 software (SPSS Inc, Chicago, USA).

## Results

We found 722 confirmed cases of COVID-19, 2213 confirmed cases of Influenza (A and B), and 482 confirmed cases of RSV infection. All were identified using nasal swab reverse transcription polymerase chain reaction (RT-PCR). Baseline characteristics, including age, sex, Charlson comorbidity score and vital signs at admission were compared between the three groups (Table 1). Age and sex were different between the groups with the patients in the RSV group being significantly older (mean 79 years, P < 0.001), and with significantly more men in the COVID-19 group (57.62%, P < 0.001). Comorbidities were presented by the Charlson score, which was significantly higher among the RSV patients (mean 5.0, P < 0.001). Regarding vital signs, body temperature was significantly higher in the COVID-19 group with a mean of 37.4 degrees Celsius (P < 0.001). Oxygen saturation at presentation was similar between the three groups.

**Table 1:** Characteristics of patients presenting with viral infections.

	COVID19	Influenza	RSV	P value
**N**	722	2213	482	
Age (years)^a^	67 (28)	73 (26)	79 (18)	P < 0.001
Sex (% males)	57.6	48.3	46.4	P < 0.001
Charlson comorbidity index^a^	3 (4)	4 (4)	5 (3)	P < 0.001
**Vital signs**				
Body temperature^a^ (℃)	37.4 (1.2)	37.1 (1.1)	37 (1.1)	P < 0.001
Heart rate^a^ (beats/min)	86 (23)	92 (26)	88 (26)	P < 0.001
Systolic BP^a^ (mmHg)	134 (30)	134 (34)	141 (38)	P < 0.001
Diastolic BP^a^ (mmHg)	76 (19)	74 (18)	74 (22.75)	P < 0.005
Oxygen saturation^a^ (%)	96 (5)	97 (26)	96 (5)	P = 0.074

^a^Continuous variables are presented as medians and inter-quartile ranges.

*COVID-19* coronavirus disease 2019, *RSV* respiratory syncytial virus, *BP* blood pressure.

**Figure 1:**
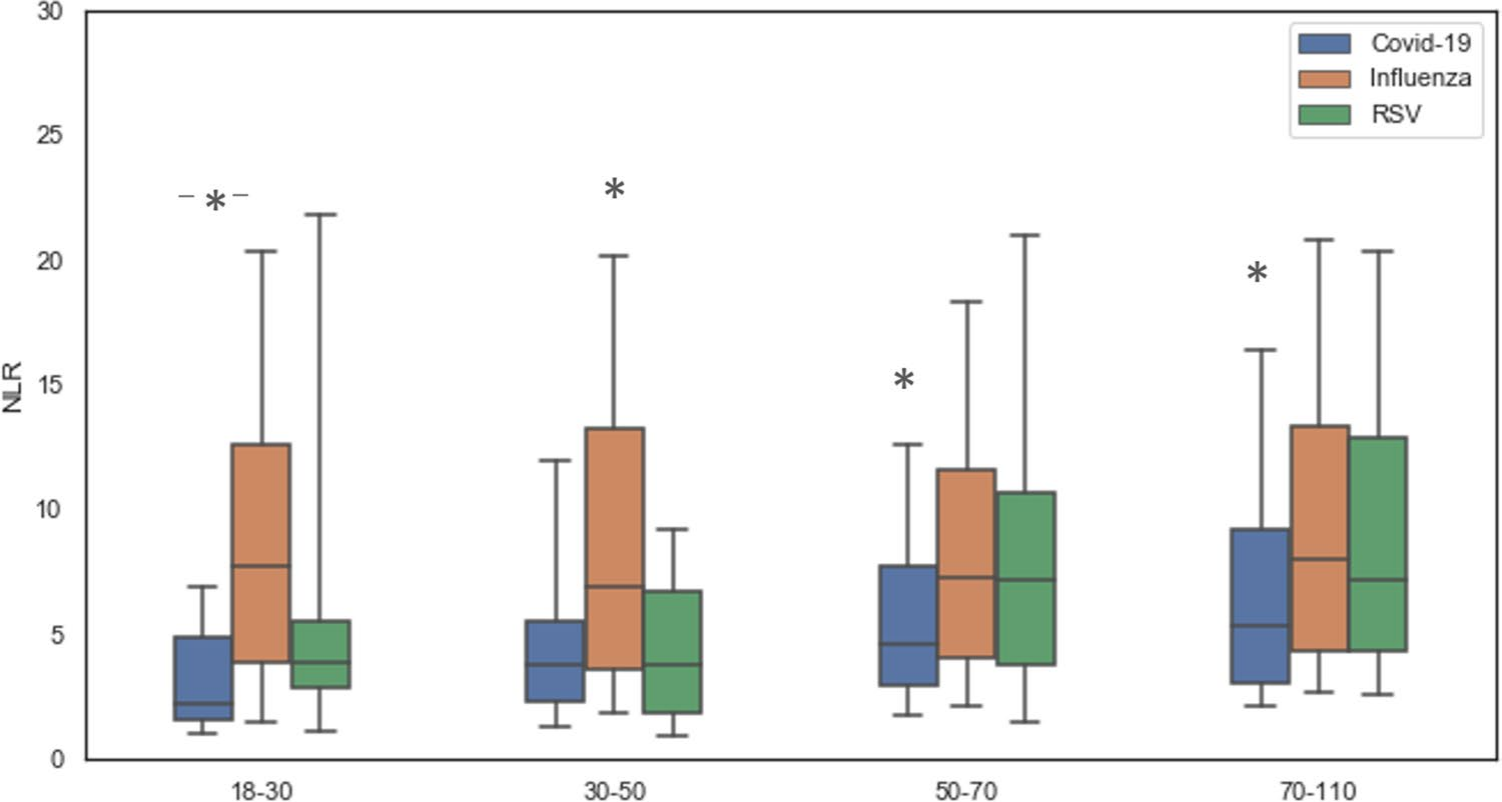
NLR levels stratified by age groups. For each age group level of NLR was compared among COVID-19, Influenza and RSV patients. The average levels are displayed by mean and SD. *NLR* neutrophil-to-lymphocyte ratio, *COVID-19* coronavirus disease 2019, *RSV* respiratory syncytial virus.

A comparison of inflammatory markers at presentation including NLR, C-reactive protein (CRP), ferritin and triglycerides is displayed in Table 2. The Influenza patient’s median NLR was significantly higher (7.54, P < 0.001), than that of the RSV (median of 6.83) and COVID-19 patients (median of 4.5). The CRP levels did not differ between the groups, but the ferritin and triglycerides levels were significantly higher in the COVID-19 group (median of 434.35 ng/mL and 123 mg/dL respectively, P < 0.001 for both). As NLR levels are age-dependent, we stratified NLR levels by age groups (18–30, 30–50, 50–70, and 70–110). Across all ages the NLR of COVID-19 patients was lower than that of Influenza patients; above the age of 50, NLR of COVID-19 patients was lower than that of both Influenza and RSV patients (Fig. 1).

**Table 2:** Inflammatory markers of patients presenting with viral infections.

	COVID19	Influenza	RSV	P value
Lymphocytes (10e3/µL)^a^	1.0 (0.8)	0.9 (0.8)	1 (0.83)	P < 0.001
Neutrophils (10e3/µL)^a^	4.9 (3.9)	6.6 (4.8)	7.1 (5.15)	P < 0.001
NLR^a^	4.5 (5.2)	7.5 (8.9)	6.8 (7.7)	P < 0.001
CRP (mg/L)^a^	57.3 (115.3)	47.7 (91.2)	50.4 (92.9)	P = 0.98
Ferritin (ng/mL)^a^	434.4 (746)	238.1 (347)	210 (364)	P< 0.001
Triglycerides (mg/dL)^a^	123 (80.3)	109 (79)	108 (73.25)	P < 0.001

^a^Continuous variables are presented as medians and inter-quartile ranges.

*COVID-19* coronavirus disease 2019, *RSV* respiratory syncytial virus, *NLR* neutrophil-to-lymphocyte ratio, *CRP* C-reactive protein.

Outcomes in our study included need for mechanical ventilation and death within 30 days of admission (Table 3). Mortality was significantly higher in the COVID-19 and RSV groups compared with the influenza group (16.1%, 13.48% and 8.8% respectively, P < 0.001). A significantly greater proportion of patients required mechanical ventilation in the COVID-19 group compared to the influenza and RSV groups (9.6%, 6.6% and 8.1% respectively; P < 0.03).

**Table 3:** Components of adverse outcomes score of patients presenting with viral infections.

	COVID19	Influenza	RSV	P value
Death in 30 days (%)	16.1	8.8	13.48	P < 0.001
Mechanical ventilation (%)	9.6	6.6	8.09	P < 0.03
Composite score^a^ (%)	19.5	12.8	18.9	P < 0.001

^a^Composite score considered positive if one of the following criteria were met: mortality, defined as death at 30 days or need for mechanical ventilation.

*COVID-19* coronavirus disease 2019, *RSV* respiratory syncytial virus, *LOS* length of stay.

For purposes of analysis, a poor outcome was defined as the presence of any of the two above—mentioned endpoints. Among the three viruses, the COVID-19 patients had the highest composite score of poor clinical outcomes, when compared to influenza and RSV patients (19.5%, 12.5%, 18.9% respectively P < 0.001).

**Figure 2:**
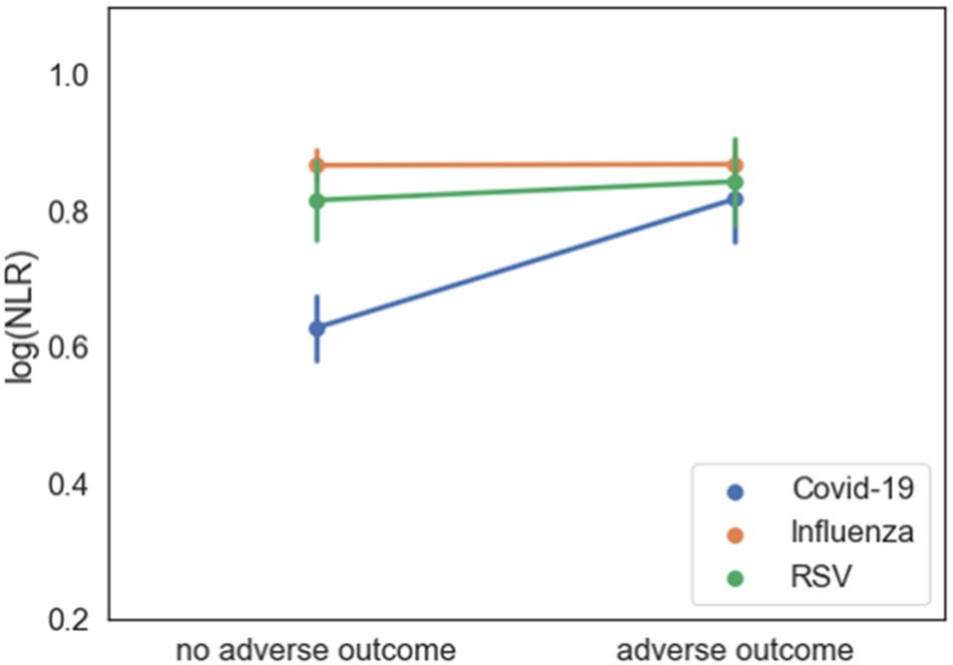
Two-way analysis of variance (ANOVA), using log-transformed NLR levels as the dependent variable, comparing the association between NLR values and adverse outcomes among COVID-19, Influenza and RSV patients. *NLR* neutrophil-to-lymphocyte ratio, *COVID-19* coronavirus disease 2019, *RSV* respiratory syncytial virus.

We compared the association between NLR values and adverse outcomes among the three groups using two-way analysis of variance (ANOVA), using log-transformed NLR levels as the dependent variable. A main effect was found for virus type: lower levels of NLR in COVID-19 in comparison to influenza and RSV (P < 0.001). Simple main effects analysis showed that in the COVID-19 group, poor clinical outcome was associated with higher NLR levels (P < 0.001), but no such association was apparent in the influenza and RSV groups (Fig. 2).

**Figure 3:**
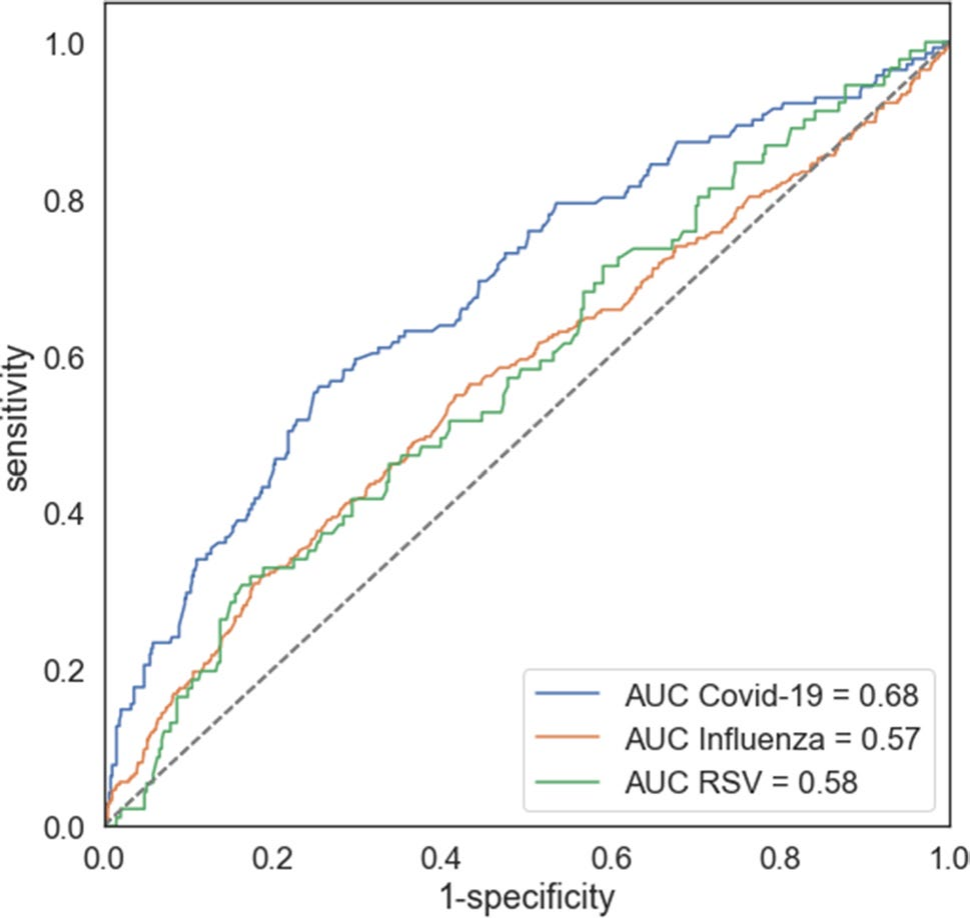
Receiver operating characteristic (ROC) curves analyses for predicting poor outcomes for COVID-19, Influenza and RSV patients. *NLR* neutrophil-to-lymphocyte ratio, *COVID-19* coronavirus disease 2019, *RSV* respiratory syncytial virus, *AUC* area under the curve.

Since higher levels of NLR were found to be associated with poor clinical outcome in the COVID-19 group, we set out to further test the discrimination ability of NLR using ROC curve analysis within each virus group (Fig. 3). The area under curve (AUC) of poor outcomes for COVID-19, Influenza and RSV was 0.68, 0.57 and 0.58, respectively. The optimal cutoff of NLR for COVID-19 was obtained from Youden’s index: NLR of 6.82. We did not further analyze the other group’s ROC curves because of the low AUCs.

**Figure 4:**
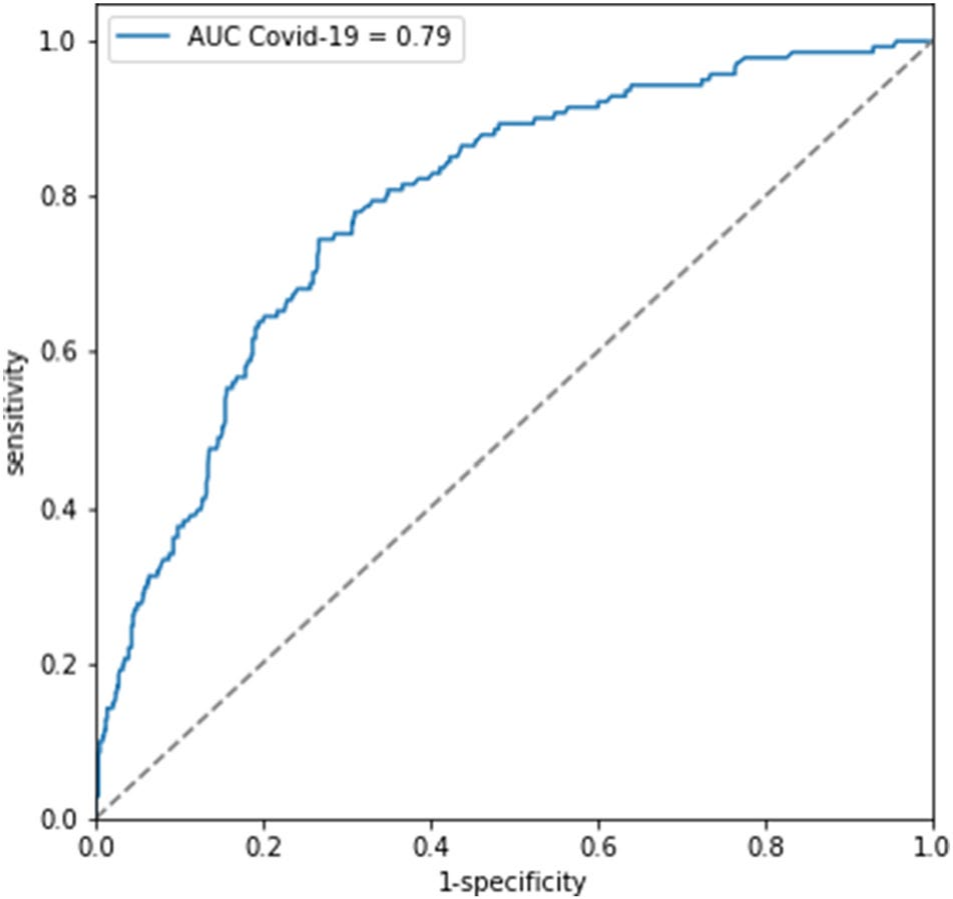
Receiver operating characteristic (ROC) curve for logistic regression model used to test the discrimination ability of NLR (above or below 6.82) as a prognostic factor of poor clinical outcome adjusted to age, sex and Charlson comorbidity index among COVID-19 patients. *NLR* neutrophil-to-lymphocyte ratio, *COVID-19* coronavirus disease 2019, *AUC* area under the curve, *CCI* Charlson comorbidity index.

Multivariate logistic regression was used in order to test the discrimination ability of NLR (above or below 6.82) as a prognostic factor of poor clinical outcome adjusted for age, sex and Charlson score. Results showed an odds ratio (OR) of 2.88 for NLR > 6.82 (P < 0.001) and a total AUC of 0.79 (Fig. 4). In this model age had an OR of 1.039 (P < 0.001) and sex was found to be non-significant in predicting a poor outcome among the COVID-19 patients (P = 0.66). Similar to high NLR, Charlson score was found to be a prognostic factor for adverse outcomes with an OR of 1.17 (P < 0.005).

### Discussion

Faced with an emerging COVID-19 pandemic, considerable efforts have been implemented in order to identify clinical and laboratory prognostic factors aiding in triage and resource allocation^40,41^.

One such factor, Neutrophils to lymphocytes ratio (NLR), has been extensively studied and yields a good predictive capacity using varying cutoffs. The immune dysregulation that characterizes COVID-19 has been implicated as a possible explanation for its strong predictive value^31^. Anticipating the winter and influenza season, we set out to compere the predictive value of NLR across three respiratory viruses; SARS-CoV-2 (The causative agent of the infection named COVID-19), Influenza (A and B) and Respiratory syncytial virus (RSV).

We compared the demographic, laboratory and clinical characteristics between these three respiratory viruses. RSV patients were significantly older (mean 79 years) with more comorbidities (expressed in a mean Charlson score of 5.0) (Table 1). Differences in mean body temperature were found to be statistically significant, with slightly higher values for COVID-19 patients, but the difference was not clinically meaningful. Interestingly, oxygen saturation at presentation was similar between the three groups. COVID-19 patients had a unique inflammatory profile, with significantly lower NLR values at presentation but with higher ferritin and triglycerides. CRP levels at presentation showed no significant difference across viruses. COVID-19 patients had higher 30-day mortality rates and a larger proportion of patients required mechanical ventilation.

Reviewing the literature we found few small—scale studies comparing laboratory parameters of adult patients with COVID-19 and Influenza^32,42,43^ and none compared with RSV. Only two studies investigated the NLR levels in COVID-19 compared to Influenza. One found no difference, and the other found that NLR was significantly lower in the COVID-19 patients (median of 2.6), as was described in our report^32,44^. In all reports, as in ours, CRP levels at admission were similar across the viruses.

Focusing on the prognostic value of NLR, we directly compared the association between NLR and a composite adverse outcome score between the three viruses. Only in the COVID-19 group poor clinical outcome was associated with higher NLR. Furthermore, when performing ROC analysis, only in the COVID-19 group NLR had a discrimination ability between patients with poor and favorable outcomes. Taken together, these results strongly suggest that NLR is a more valuable prognostic marker in COVID-19 compared to Influenza and RSV. This is opposed to former reports, which identified the role of NLR as a predictor of poor prognosis in Influenza patients^15–18^.

The application of NLR as a prognostic factor in COVID-19 has been extensively studied. A high NLR implies an aberrant immune response, with increased neutrophils and decreased lymphocytes^31^. Neutrophil production can be triggered by virus-related inflammatory factors, such as interleukin-6 and interleukin-8, tumor necrosis factor-α, granulocyte colony stimulating factor, and interferon-γ. Lymphopenia is common in COVID-19, as a result of direct cytokine—induced inhibition^45^. The massive over—production of these cytokines, or "cytokine storm", can result in acute respiratory distress syndrome (ARDS), the hallmark of severe cases of COVID-19^46^. In one cohort, all COVID-19 patients with ARDS had an aberrant immune response [either macrophage activation syndrome (MAS) or "immune paralysis" (depletion of HLA-DR expression)]^47^. These patients had a sustained production of IL-6 and TNF-α, which was unique when compared to a retrospective cohort of influenza H1N1 2009 patients. Moreover, in vitro, higher levels of IL-6, TNF-α and IL1-β were released from monocytes derived from COVID-19 patients compared to H1N1 2009 patients.

In contrast to previous reports, our results did not demonstrate a prognostic value for NLR in influenza patients. This discrepancy may possibly be attributed to the small number of patients reported in previous studies.

In RSV infection, NLR has not been hitherto studied. Current understanding of the immune response to RSV infection holds that severe cases are initially associated with neutrophilia and lymphopenia and thus would be expected to be associated with a high NLR.

Following the neutrophilic response, RSV infection is associated with a pulmonary CD8 T-cell response and lymphocytes levels rise, coinciding with viral clearance^48^. Thus, theoretically, a low NLR would be expected to imply a favorable prognosis. Nonetheless, in our real-life, large cohort of RSV patients, NLR at admission did not have prognostic value.

In order to determine the applicable threshold for high NLR we used a ROC curve. The optimal NLR threshold at 6.82 had the highest sensitivity and specificity for differentiating favorable and poor outcome. This result is in concordance with previous studies, in which the proposed optimum cutoff values ranged from 3 to 6. Two studies stratified the prognostic capacity of NLR by age^21,49^. In our study, a logistic regression model was used in order to test the discrimination ability of NLR (above or below 6.82) in predicting clinical outcomes adjusted to age, sex and Charlson comorbidity score. Our results show an OR of 2.882 for NLR and a total AUC of 0.79 (Fig. 4). This reinforces the conclusion that NLR is a valuable prognostic marker in COVID-19 patients, even after stratification according to sex, age and comorbidities.

Our study has several limitations. First, since it is a retrospective observational study, subject selection bias was unavoidable. Second, the COVID-19 pandemic resulted in the referral of patients with minimal upper respiratory symptoms and general good health to the ER. Thus, the COVID-19 cohort included a large subset of relatively healthy subjects, with a mild disease, skewing NLR levels towards a lower baseline. RSV and influenza patients with a correspondingly mild disease severity were probably treated in the community (in which case their data is absent) or alternatively presented to the ER later in the course of their disease, at a worse clinical stage. Notably, such later presentations may have created an additional limitation, the timing of the first complete blood count, which was performed at an earlier stage among COVID-19 patients.

Previous research has raised the possible utility of the cycle threshold (CT) value as a prognostic factor in COVID-19 patients^50^. While this data was not available in the current study, future investigation comparing, and possibly combining, the NLR with the CT values may prove useful in fine-tuning the prognostic evaluation of COVID-19 patients.

In conclusion, we present the first large scale study to compare laboratory markers and outcome between COVID-19, influenza and RSV. We show that COVID-19 patients had a lower NLR at admission. We calculated a NLR cutoff of 6.82 which was able to differentiate between poor and favorable outcome. Finally, when comparing the three viruses, only in COVID-19 NLR had a prognostic value. Thus, during the upcoming winter months, physicians faced with the daunting challenge of treating and triaging patients presenting with acute respiratory infections, may benefit from the correct implementation of this readily accessible tool in their clinical decision-making in COVID-19 patients.
